# Amphiphilic Oxygenated Amorphous Carbon-Graphite Buckypapers with Gas Sensitivity to Polar and Non-Polar VOCs

**DOI:** 10.3390/nano9091343

**Published:** 2019-09-19

**Authors:** Shahin Homaeigohar

**Affiliations:** Nanochemistry and Nanoengineering, Department of Chemistry and Materials Science, School of Chemical Engineering, Aalto University, Kemistintie 1, 00076 Aalto, Finland; Shahin.homaeigohar@fau.de

**Keywords:** carbon nanofilament, amphiphilicity, gas sensing, volatile organic compounds (VOCs)

## Abstract

To precisely control the emission limit of volatile organic compounds (VOCs) even at trace amounts, reactive nanomaterials of, e.g., carbon are demanded. Particularly, considering the polar/non-polar nature of VOCs, amphiphilic carbon nanomaterials with a huge surface area could act as multipurpose VOC sensors. Here, for the first time, a buckypaper sensor composed of oxygenated amorphous carbon (a-CO_x_)/graphite (G) nanofilaments is developed. Presence of the oxygen-containing groups rises the selectivity of the sensor to polar VOCs, such as ethanol and acetone through formation of hydrogen bonding, affecting the electron withdrawing ability of the group, the hole carrier density, and, thus, the resistivity. On the other hand, the electrostatic interactions between the toluene aromatic ring and the π electrons of the graphitic crystals cause a formation of charge-transfer complexes, which could be the main mechanism of high responsiveness of the sensor towards non-polar toluene. To the best of my knowledge, an amphiphilic carbon nanofilamentous buckypaper has never been reported for gas sensing, and my device sensing polar/non-polar VOCs is state of the art for environmental control.

## 1. Introduction 

The pollution of water streams or air is one of the most critical concerns shaping world society in the coming century [1,2]. In this regard, the quality of air is progressively deteriorating in metropolitan areas. Introduction of chemicals, particulates, or biological materials into the atmosphere via different sources brings about discomfort, disease, or death to humans [3]. In addition to the outdoor air pollutants, the indoor air pollutants, such as volatile organic compounds (VOCs), are also detrimental to human health, comfort, and productivity. VOCs are among the major pollutants that are inhaled daily [4,5]. Inhalation of some of these compounds could lead to sick building syndrome (SBS), causing mucous membrane irritation, headaches, and fatigue [5,6,7,8,9] and also to other health problems, even to cancer (e.g., caused by formaldehyde, acrolein [10]). Thus, remediation and control of the air pollutants based on novel cost/energy efficient materials and technologies is crucially important. Nanotechnology could be an optimum solution for not only removal of hazardous VOCs [11], but also precise control of their emission limit, even at trace amounts [12].

With respect to gas sensors, there is a need for simple, sensitive, and stable electronic sensors, allowing for trace detection of gases, replacing the current expensive, bulky, and complicated sensing equipment. In this regard, reactive nanomaterials with a huge surface area, optimum porosity, and desirable pore size that drive the chemical/physical adsorption of gases are of paramount importance [13]. Among the various nanomaterials proposed for construction of gas sensors, carbon nanomaterials have been appealing and attracting wide research interests. Thanks to a combination of excellent detection sensitivity and promising transduction properties, carbon nanomaterials are potential candidates for the next generation of autonomous sensor technology. In 0–2 dimensional (D) carbon nanostructures, most of the atoms are exposed to the environment, thus offering a high specific surface area and enabling notable sensitivity [14,15,16]. Advantageous over the polymeric counterparts, they are not sensitive to swelling, high temperatures, or chemically harsh conditions [17,18,19,20]. Moreover, as compared to the conventional metal oxide semiconductor (MOS)-based sensors operating at a high temperature, the carbon based sensors show good analytical sensitivity at room temperature [21,22]. Therefore, higher safety, i.e., a reduced risk of explosion in the presence of combustible gases and less energy consumption, are guaranteed [21,23]. In the past years, for gas sensing applications, carbon nanotubes have been most widely investigated among carbon nanomaterials. The other attractive options are graphene and graphitic carbon nitride, and the research community has started to explore their potential in this relevance [14,24,25]. As another fascinating type of carbon nanomaterial, carbon nanofibers (CNFs) made from electrospun polyacrylonitrile (PAN) nanofibers have also been spotlighted for the construction of sensors and biosensors. This motivation stems from their outstanding characteristics, including their 1D nanostructure, functionalization ability, electrochemical properties, mechanical flexibility, and biocompatibility [26]. 

Mao et al. [27] have shown that the electrochemical activities of the CNF-based webs, thereby their sensing function, can be tuned via controlling their density of electronic states (DOSs) near the Fermi level. The DOS of CNFs is dependent on their nanosized graphite concentration, which can be tailored via manipulation of the carbonization conditions. As we recently showed [28,29,30], a high graphitization temperature of 1250 °C brings about a hybrid nanostructure of graphite and amorphous carbon, thus an optimum DOS state. In contrast to the large graphite regions, the nanosized regions with many edge-plane sites, as we observed in our CNFs, offer a remarkably higher DOS near the Fermi level because of the overlap between the valence and conduction bands [31]. The higher the graphitization temperature (e.g., 1250 °C in our study), the larger the concentration of nanometric graphite clusters in the CNF webs, and thus more notable the DOS will be.

**Figure 1:**
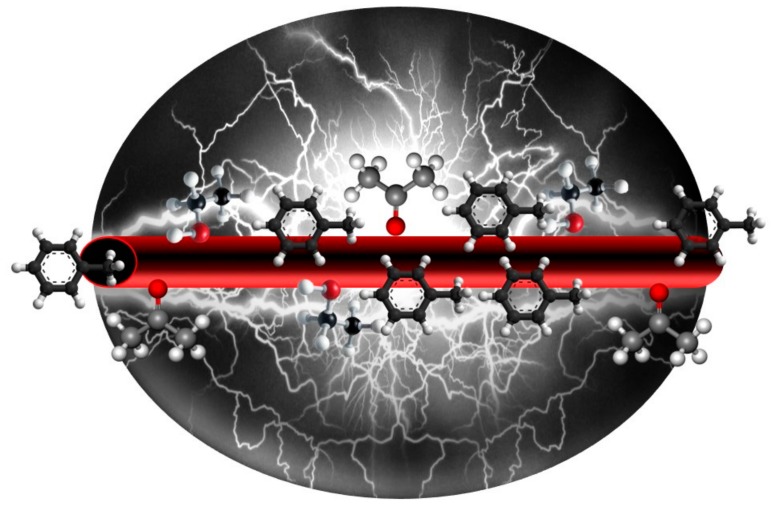
Electrically conductive oxygenated amorphous carbon (a-CO_x_)/graphite (G) (a-CO*_x_*/G) nanofibers able to sense polar (here, ethanol and acetone) and non-polar (here, toluene) volatile organic compounds (VOCs).

In a very limited number of studies on CNFs for sensing applications, the electroanalytical activities of the CNF sensor have been modified by employing an additional active component, such as metal nanoparticles [32,33,34,35]. Inclusion of the secondary elements is not straight forward and involves a complicated chemistry and thus raises the related costs and hampers scalability of the process. In contrast, the above-mentioned strategy, relying on optimization of the electrochemical activity of a CNF sensor by manipulating its carbonization treatment, excludes the need for a secondary element and its related chemical processes. As an extra advantage, as we recently showed [28], presence of an amorphous carbon phase, along with the graphitic nanodomains, enables oxidation of the CNFs and thus provides an amphiphilicity effect, beneficial for detection of polar and non-polar VOCs. Simultaneous detection of various VOCs, particularly with different polarity levels, is crucially important and assures versatility and wide applicability of the sensor [36,37,38,39,40,41,42]. However, in contrast to our approach, such a possibility i.e. sensing of several analytes with different physicochemical nature is typically achieved through intricate chemical pathways or involves inclusions to confer the sensing material with the desired reactivity or to engender detectability by, e.g., dyes or plasmonic materials [43,44]. On the other hand, the detection principle itself could be more challenging versus a simple electrical resistivity measurement and could necessitate employment of sophisticated set-ups. Here, for the first time, interesting properties of amorphous carbon/graphite hybrid nanofilaments and their application in gas sensing and even removal of various polar and non-polar VOCs are demonstrated, Figure 1. 

## 2. Experimental

### 2.1. Materials

As the carbon nanofiber precursor, PAN, with the molar mass of 200,000 g.mol^−1^, was purchased from Dolan GmbH (Germany). The solvent *N*,*N*-dimethylformamide (DMF) was obtained from Merck (Germany). All the materials were used as received.

### 2.2. Sample Preparation

The PAN nanofibrous mats as the precursor of carbon nanofibrous membranes were produced by an electrospinning method. Briefly, a prepared PAN solution (8 wt.% in DMF) was fed with a constant rate (0.8 ml·h^−1^) into a needle by using a syringe pump (Harvard Apparatus, Holliston, MA, USA). By applying a 15 kV voltage (Heinzinger Electronic GmbH, Rosenheim, Germany), PAN was electrospun on an aluminum foil located 25 cm above the needle for 8 h. 

**Figure 2:**
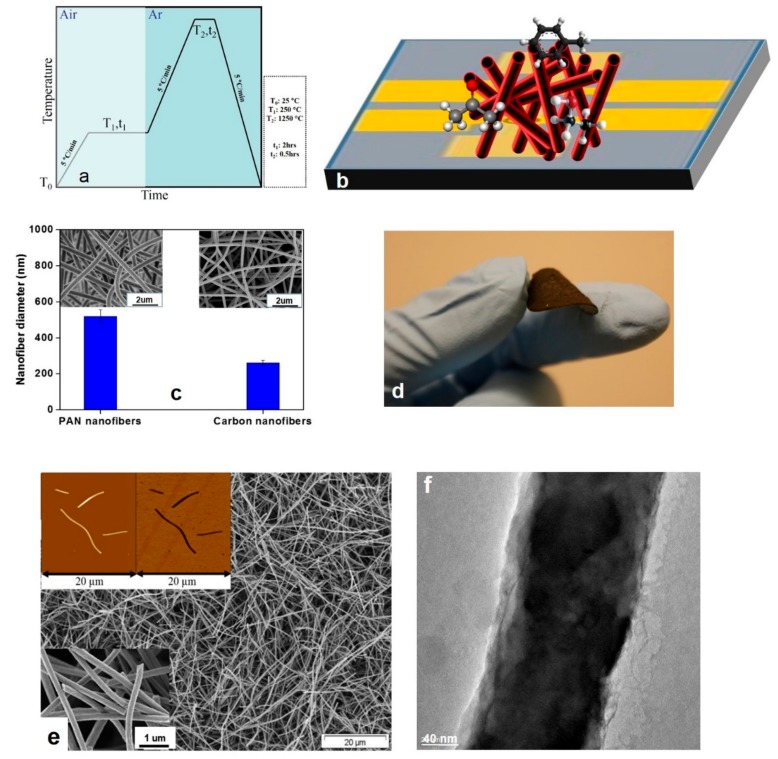
(**a**) The step-wise production process of a-CO_x_/G nanofibers; (**b**) the schematic illustration of the gas sensing set-up comprising a glass substrate overlaid by gold contact electrodes, where the nanofilaments sit and are exposed to the gas analytes; (**c**) morphology and dimensions of the a-CO_x_/G nanofibers (the nanofibers’ diameter shrinks within the course of the graphitization process); (**d**) the camera image shows the flexibility of the buckypaper; (**e**) morphology and dimensions of the chopped a-CO_x_/G nanofilaments (the inset pictures are the atomic force microscopy (AFM) micrographs showing morphology and dimension of individual nanofilaments (upper left) and the SEM micrograph showing morphology of the nanofilaments at a high magnification (lower left)); (**f**) TEM image indicates the presence of two distinct regions distinguished by their different color intensity. The darker regions imply the aligned, graphitic, and dense arrangement of carbon elements, while the brighter regions are related to sparse and haphazard amorphous carbon.

After peeling off from the aluminum foil, as the oxidative stabilization step, the electrospun nanofibrous mats were put in a furnace (Linn Elektro Therm GmbH, Bad Frankenhausen, Germany, max T = 1250 °C) and heated in air at 250 °C for 2 h. Subsequently, the air-oxidized membranes were graphitized in argon atmosphere at the temperature of 1250 °C for 30 min and then cooled fast down to the ambient temperature. The heating and cooling rates were 5 °C.min^−1^. The whole process of making carbon nanofibers is seen in Figure 2a. As mentioned earlier, the process led to the formation of biphasic amphiphilic oxygenated amorphous carbon (a-CO_x_)/graphite (G) (a-CO*_x_*/G) nanofibers.

The obtained a-CO*_x_*/G nanofibrous membrane (0.03 gr) was immersed in 10 ml distilled water and ultrasonicated for 2 min at an amplitude of 20%. The ultrasonication process chops the a-CO*_x_*/G nanofibers, leading to formation of a homogenous aqueous suspension of the chopped a-CO*_x_*/G nanofibers, which was cast over a circular poly(*p*-phenylene sulfide) (PPS) technical non-woven (diameter = 3.5 cm). The membrane was left to be dried in air overnight.

### 2.3. Structural Characterizations

The morphology of the chopped a-CO*_x_*/G nanofibers (hereafter called nanofilaments) was characterized by a scanning electron microscope (SEM) (LEO 1550VP Gemini from Carl ZEISS, Jena, Germany) and atomic force microscopy (AFM) (MultiMode^TM^ Atomic Force Microscope from Bruker AXS, Madison, WI, USA). The diameter of the nanofibers was determined from the SEM images using the Adobe Acrobat v.07 software (Adobe Inc., San Jose, CA, USA). The surface area of the nanofibers and nanofilaments was measured by a Micromeritics ASAP 2020 (Micromeritics Instrument Corp., Norcross, GA, USA), according to the standard multi-point Brunauer–Emmett–Teller (BET) technique, using N_2_ as the analysis gas. 

Structural analyses of the PAN and a-CO*_x_*/G nanofibers were carried out at room temperature using an X-ray diffractometer (XRD3000TT, RICH. SEIFERT & Co GmbH, Ahrensburg, Germany) with Cu-Kα radiation (= 0.15418 nm). In addition, chemical surface analysis of the PAN and a-CO*_x_*/G nanofibers was performed by Attenuated Total Reflection Fourier-transform infrared spectrometry (ATR-FTIR) (ALPHA (ATR-Ge, ATR-Di) from BRUKER Optik GmbH, Ettlingen, Germany). 

### 2.4. Measurement of Gas Responsiveness 

For the gas sensing measurements, the a-CO*_x_*/G nanofilaments were first cast onto a glass slide overlaid by gold contact electrodes, as in Figure 2b. Such an assembly was then exposed to three VOCs of acetone, ethanol, and toluene (100 ppm) at room temperature. Any variation in the electrical resistivity of the buckypapers at room temperature was recorded in a given duration through a programmable electrometer (Keithley 6517B). During the measurement, upon reaching a steady and stable electrical resistance, the injection of VOC gas was turned off and the electrical resistance’s recovery was observed. The gas response of the sensor is expressed as the normalized resistivity calculated by the following Equation (1):

(1) $$S\left(\%\right)=\frac{\Delta R}{R_{0}}\times 100=\frac{R_{g}-R_{0}}{R_{0}}\times 100$$

where *R*_0_ is the resistance at the onset of the experiment and *R*_g_ is the resistance measured upon exposure to the VOC gas.

For the sake of comparison and to evaluate the effect of graphitization on toluene sensitivity (removal/adsorption), the samples were further chemically functionalized to achieve a higher density of polar oxygen-containing groups. To functionalize the samples, citric acid (300 mg) was added to the aqueous suspension of a-CO*_x_*/G nanofibers. The remaining preparation steps of ultrasonication and casting were the same as those performed for the neat samples. 

## 3. Results and Discussion

The a-CO_x_/G nanofibers were made through electrospinning of a polymer precursor, i.e., PAN and its subsequent stabilization and graphitization at the high temperature of 1250 °C, as shown in Figure 2a. This method is simpler and cheaper than the counterpart production methods, such as the vapor growth method and plasma enhanced chemical vapor deposition [45,46,47]. Moreover, it can be used to make a web structure. When used as an electrode, there is no need to a second processing step involving a binder and an electric conductor, e.g., carbon black. Thus, the electrospun webs are promising in terms of ease of handling, an increase in the energy density, thanks to a large specific surface area, enhanced conductivity, due to an increased density of the contact points, and inexpensive preparation of the electrodes [48].

### 3.1. Morphological Characteristics

Morphology of the a-CO_x_/G nanofibers made by graphitization of PAN nanofibers is seen in Figure 2c. An obvious decrease of the diameter is observed during the graphitization process, as measured based on SEM images.

To severely graphitize the carbon nanofibers as a prerequisite for the removal of impurities, an optimum interaction with VOCs [49], and also enhancement of the electrical conductivity [50] for gas sensing applications, they should be carbonized at high temperatures. On the other hand, as we previously demonstrated [28], carbon nanofibers must be rapidly quenched to preserve a fraction of amorphous carbon for a subsequent oxidation and thus surface decoration with oxygen-based functional groups. Despite the merit of the as-developed amphiphilic nanostructure for interaction with plar/non-polar gases, this sort of carbonization makes the nanofibrous mat drastically brittle, thereby difficult to handle [17,18,51,52,53]. Here, we benefitted from this drawback and chopped the nanofibers to enhance their surface area for a higher interaction with VOCs. Subsequently, the chopped nanofilaments were cast as a buckypaper on a commercial non-woven of PPS through a vacuum suction process. As seen in Figure 2d, the obtained buckypaper is totally flexible and comprises arbitrarily arranged nanofilaments. The image of the resulting structure is seen in Figure 2e. As deduced from this image, the sensor is highly porous, thus interactive with VOCs. Moreover, as seen in the inset Figure 2e, the nanofilaments as the constituents of the sensor are several µm in length and ~260 nm in breadth. The TEM image in Figure 2f shows the arrangement of graphitic zones along with amorphous carbon domains.

### 3.2. Structural Characteristics

**Figure 3:**
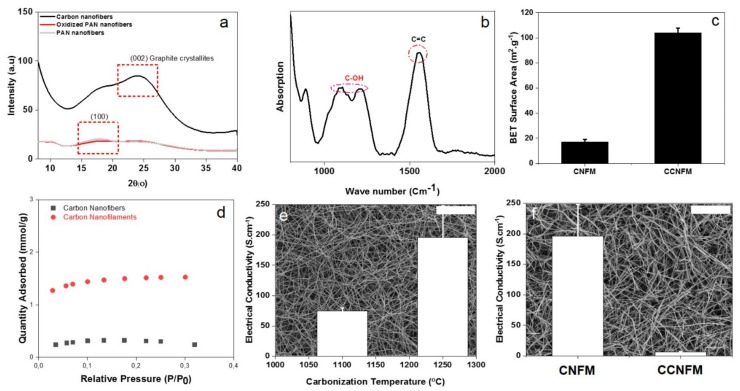
Structural properties of the a-CO_x_/G nanofilaments. (**a**) XRD verifying formation of graphite crystallites, along with a-CO_x_ domains; (**b**) ATR-FTIR showing the surface chemistry of a-CO_x_/G nanofilaments; (**c**) Brunauer–Emmett–Teller (BET) surface area measured for the carbon nanofilament-based membrane (chopped carbon nanofilament membrane (CCNFM)) versus the carbon nanofiber-based membrane (carbon nanofiber membrane (CNFM)), stressing the effect of the chopping process on the enhancement of the surface area; (**d**) N_2_ adsorption isotherm for the CNFM and CCNFM, implying larger adsorption of nitrogen molecules onto the CCNFM versus the CNFM, due to its more extensive surface area. (**e**) Influence of the carbonization temperature on electrical conductivity of carbon nanofibers (CNFs); (**f**) electrical conductivity of the CCNFM versus that of the CNFM. The large difference between the conductivity of the CNFM and CCNFM implies that the chopping process declines the conductivity by lowering the continuity and cross links of the fibers, as witnessed by the inset SEM images (scale bars represent 20 µm).

XRD could be an informative tool to indicate the macromolecular and crystalline structure of the a-CO_x_/G nanofilaments. As seen in Figure 3a, the PAN nanofibers show a more obvious diffraction peak located at 2θ = 17°, representing the X-ray reflections of the (100) crystallographic planes in PAN [54]. After stabilization, as seen for the oxidized PAN nanofibers, the (100) peak disappears, implying that all the nitrile groups react to form a ladder-like structure for the stabilized PAN nanofibers [54]. Eventually, after thermal annealing, a diffraction peak appears at 2θ = 26°, which is attributed to a (002) crystallographic plane of graphite crystallites [54].

The Bragg and Scherrer equations (Equations (2) and (3)) were implemented to calculate the average interplanar spacing “d(002)” and crystallite size parameter “Lc” as follows [54]:

(2) $$d_{\left(002\right)}=\frac{\lambda }{2\sin\theta }$$

(3) $$L_{c}=\frac{0.9\lambda }{\beta \cos\theta }$$

where *λ* is the wavelength of the X-ray, *θ* the diffraction angle, and *β* the width of the diffraction peak measured at half of its height in radian.

The graphite nanosheets showed a d(002) of 0.35 nm and a crystallite size parameter “Lc” of 0.59 nm; the former parameter is slightly larger than that for the natural graphite (0.33 nm), i.e., a less ordered graphitic structure is formed due to presence of the oxygenated amorphous carbon domains [54,55].

ATR-FTIR (Figure 3b) indicates the chemical composition of the a-CO_x_/G nanofilaments used in the sensor structure. The strong peak emerging at 1589 cm^−1^ is related to the C=C groups formed by the aromatization process, taking place during the thermostabilization of PAN nanofibers. This characteristic peak represents the non-polar graphitic regions [56,57]. The other main characteristic group seen at 1000–1300 (2 bands) is about the C-OH bond and is representative of a-CO_x_ domains [58]. Thus, the ATR-FTIR analysis of the nanofilaments implies coexistence of a-CO_x_ and graphite or amphiphilicity of the structure. 

As we recently showed [28], the carbonization temperature has a notable impact on the graphitization degree of CNFs and the removal of impurities, such as nitrogen and oxygen. This effect subsequently enhances electrical conductivity of the membrane, as seen in Figure 3e. On the other hand, to induce amphiphilicity, the CNFs are rapidly cooled down to preserve a minor fraction of amorphous carbon, which could be subsequently oxidized by the oxygen separated during the annealing. The presence of the oxygen-containing groups rises the selectivity of the sensor to polar VOCs through the formation of hydrogen bonding. This interaction reduces the electron withdrawing effect of the oxygen containing groups, thus reduces the hole carrier concentration in the sensing material. On the other hand, pure graphite as a p-type semiconductor guarantees selectivity to non-polar gases. However, as mentioned earlier, such a high temperature annealing makes the a-CO*_x_*/G nanofibers significantly brittle and difficult to handle [17,18,51,52]. In order to overcome the latter bottleneck, the brittleness of the nanofibers was employed to make a buckypaper membrane consisting of a-CO*_x_*/G nanofilaments and increase the reactive surface area for interaction with gas pollutants. This increment in the surface area was verified via the BET measurements and the corresponding high affinity adsorption isotherms, Figure 3c,d, implying a significant potential for exposure of the carbon nanofilaments to the gas streams. However, as shown in Figure 3f, this structural change can lead to significant loss of conductivity compared to the continuous nanofibrous state. Despite such a large decline in conductivity (6 S.cm^−1^), it is still comparable to that of some conductive carbon-based fibers, such as graphene fibers (10 S.cm^−1^) [59], rGO fibers (~2–3 S.cm^−1^) [60], and PAN nanofibers carbonized at 900 ºC (~1–2 S.cm^−1^) [61].

### 3.3. Gas Sensing Characteristics

Thanks to the huge reactive surface area of the carbon nanofilaments, a-CO*_x_*/G buckypaper is assumed to perform as a gas purifier able to remove VOCs. This ability was probed via tracking the changes of resistivity of the a-CO*_x_*/G buckypaper upon adsorption of VOCs. On the other hand, possessing semiconductor graphite nanofilaments, the buckypaper could also act as a gas sensor. It is noteworthy that, for such an application, to date, researchers have concentrated mainly on n-type materials, while p-type semiconductors, such as carbon materials, have been scarcely studied [21,62]. 

The gas sensing performance was investigated by monitoring resistivity of the samples when exposed to three VOCs of ethanol, toluene, and acetone. VOCs are of the most significant environmental pollutants, which can be found in soil and atmosphere [63]. Among them, aromatic VOCs, such as toluene, could be human carcinogen and could lead to different forms of cancer, including hematopoietic and lymphatic cancers [63]. Despite the importance of detection of these compounds, so far, research on the sensors able to detect aromatic VOCs compared to those on other gases, such as NO, CO, H_2_, and NH_3_, have been scarce [63,64]. 

**Figure 4:**
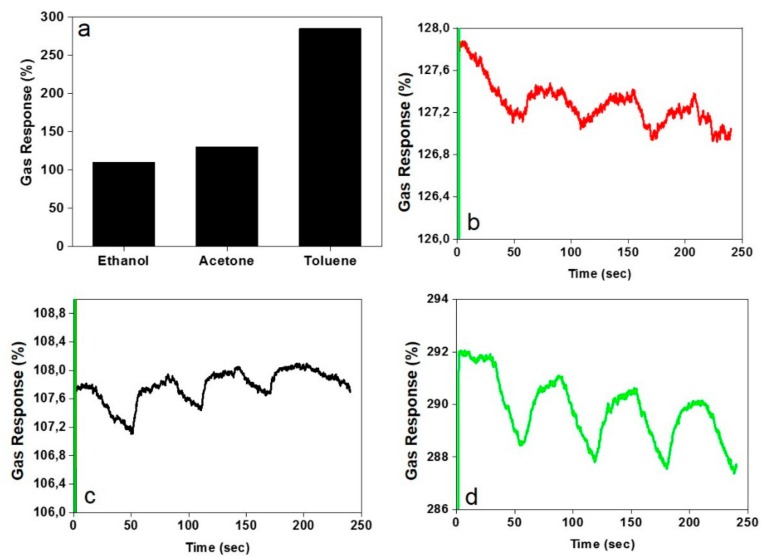
(**a**) Volatile organic compound (VOC) gas response of the a-CO*_x_*/G buckypapers; (**b**–**d**) the recovery property of the a-CO*_x_*/G buckypapers exposed to ethanol, acetone, and toluene, respectively.

Figure 4a shows the results of the electrometry measurement on the a-CO*_x_*/G buckypaper exposed to the gases, i.e., the response and recovery features. The buckypaper shows a high response to all the VOCs examined, especially to toluene. The resistivity of the samples could increase by 108–285% upon exposure to the VOCs, implying an optimum adsorption of the harmful gases to the a-CO*_x_*/G buckypaper, thereby air purification capability. 

In terms of the response time, all the samples regardless of the type of gas show an instantaneous response (less than 1 second), which is extraordinarily quick. The magnitude of the recovery property of the samples for the gases follows the order of: Ethanol < acetone < toluene. However, as shown in Figure 4b–d, the recovery is not significant, which could be interpreted as a stable adhesion of gas to the nanofilaments and a promising potential of the buckypaper as a gas purifier usable in safety equipment, such as gas masks. Needless to say, in case of heating, the buckypaper would be fully recovered for a new usage as a gas purifier.

The oxygen ionosorption is assumed to be the main gas-sensing mechanism of the a-CO*_x_*/G buckypaper. The graphite nanofilaments are considered as p-type semiconductors in which the major carriers are holes in the valence bands. When the nanofilaments are exposed to air, the oxygen molecules are absorbed onto the surface in the form of chemisorbed oxygen ions (such as O_2_^−^, O^−^ and O^2−^) [62,65], leading to the formation of a surface accepter-level and a hole accumulation layer. In the presence of reducing gases, such as ethanol, and through interactions of the gas and the surface chemisorbed oxygen ions (the following reactions), electrons are released [62]:

C_2_H_5_OH + 6O^−^ ⇔ 2CO_2_ + 3H_2_O + 6e^−^ (4)

C_2_H_5_OH + 6O^2−^ ⇔ 2CO_2_ + 3H_2_O + 12e^−^ (5)

The electrons are then recombined with the holes leading to an increase in the resistivity of the samples:

e^−^ + h^.^ ⇔ null (6)

The above-mentioned mechanism is also the case for acetone and toluene [65].

The a-CO*_x_*/G nanofilaments owing to a huge specific surface area are able to absorb large amounts of gas molecules [62]. Since carbon is a p-type semiconductor, the major carrier holes transmit inside the materials under the influence of an external electric field. Such a hole transmission can have a significant effect on the response and recovery properties of the sensor. In nanofibers different with the bulky materials, the hole accumulation layers could overlap with each other along the fiber direction, thereby form continuous hole transfer channels. Hence, the grain boundary barriers as seen in bulky materials are less influential on the transmission of the carriers along the nanofibers and this results in the quick carriers’ transmission, i.e., a very quick, high, and optimum response to the gas [62]. 

As mentioned earlier, the a-CO*_x_*/G nanofilaments possess polar functional groups of hydroxyl. Accordingly, polar organic gases, such as acetone and ethanol, can react directly to the nanofilaments. In addition to the above-mentioned mechanism involving the surface chemisorbed oxygen ions, adsorption of the polar organic molecules to the surface of the nanofilaments could alter electronic properties of the semiconductor graphite nanofibers through a charge transfer mechanism [66]. Therefore, the organic gas with the higher dipole moment, i.e., acetone (2.91 Vs. 1.60 for ethanol) can stimulate a higher responsiveness by the sensor than ethanol does. Possessing electron withdrawing groups of carbonyl induces an electron pulling effect on the nanofilaments during the exposure, leading to a higher responsiveness [67]. This behavior is influenced by the presence of oxygen functional groups within the amorphous carbon domains. Formation of hydrogen bonding between the functional groups and polar VOCs increases electrical resistance due to a decline of the electron-withdrawing force of the groups, resulting in a smaller hole carrier density in the p-type material [68]. In general, the oxygen-containing groups on a CNF pull electrons away from carbon, due to the stronger electronegativity of oxygen (3.5 eV) than carbon (2.5 eV) [68]. Such a behavior has been reported for the carbon nanotubes functionalized with fluorine, a strong electron-withdrawing element which also enhances the hole carrier density on the tubes [69].

In contrast to acetone and ethanol, toluene is non-polar [67] but stimulates the highest responsiveness by the sensor. It is assumed that toluene can also react to the nanofilaments but mostly to the non-polar parts, i.e., the dominant graphitic parts. The attractive and electrostatic interactions between toluene’s aromatic ring and the π electrons of the graphitic microcrystals and thereby formation of charge-transfer complexes among aromatic electrons of toluene molecules and the edges and lateral faces of the graphite surface could be the main mechanism for the high responsiveness of the sensor towards toluene [70]. 

**Figure 5:**
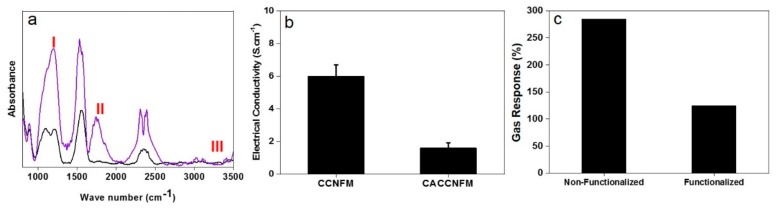
(**a**) ATR-FTIR showing the surface chemistry of a-CO_x_/G nanofilaments as non-functionalized (black line) versus citric acid (CA)-functionalized (blue line) (I, II, and III represent C-O-C, C=O, and OH groups, respectively); (**b**) electrical conductivity of the carbon nanofilament-based membrane (buckypaper) as non-functionalized versus CA-functionalized; (**c**) toluene gas response of the a-CO*_x_*/G buckypapers as CA-functionalized and non-functionalized.

To evaluate the hypothesis of interaction of toluene mainly with non-polar and highly graphitic domains of the nanofilaments, a group of the buckypapers were chemically functionalized (by a citric acid (CA) treatment). As shown in Figure 5a, the decreased intensity of the characteristic peak of −OH (at 3304 cm^–1^) and emergence of new peaks of C═O and C–O–C (at 1720 and 1196 cm^–1^) imply that CA has been successfully grafted onto the CNFs [71]. Possessing the functional groups of hydroxyl and carboxyl [72], the CA-functionalized nanofilaments show a less electrical conductivity compared to the non-functionalized ones, as shown in Figure 5b. As seen in Figure 5c, the CA-functionalized nanofilaments offer less gas response as the increase of resistivity. This behavior could imply that the presence of surface functional groups leads to less toluene adsorption and removal. This finding once more emphasizes the importance of using a high carbonization temperature, not only for purification applications and adsorptive-based removal of organics, but also for a gas-sensing technology.

## 4. Conclusions

We previously demonstrated that graphitization of polymeric nanofibers at a very high temperature followed by a fast quenching leads to formation of a biphasic, amphiphilic carbon nanofiber system. Such carbon nanofibers are optimally adsorptive to both polar and non-polar VOCs, thus could be employed for the removal of hazardous organic gases and also for a gas sensing purpose. While the polar VOCs (here, ethanol and acetone) interact with the polar oxygen-containing groups of the nanofilaments, the non-polar VOCs (here, toluene) are firmly adsorbed onto the graphitic domains. The latter was confirmed through a CA-functionalization approach that could tune functionality (polarity), thus the interaction level of the nanofilaments and toluene. The response time of the nanofilaments to the gases was extremely fast (less than 1 second). Yet, their recovery property was not perfect, implying the strong interaction of the gases with the nanofilaments. This behavior can be interpreted as the potential of the system for gas adsorption (purification).

To the best of my knowledge, this work is the first report on the design of an amphiphilic buckypaper-shaped graphite nanofibrous membrane able to perform as an air purifier and a polar/non-polar gas sensor concurrently. These findings are worthwhile considering the necessity of development of new materials addressing the current environmental pollutions either in air or water while being energy and cost efficient.
